# Supplementary figures and images for: Quantifying fish range shifts across poorly defined management boundaries
Source: PLoS One. 2023 Jan 11;18(1):e0279025. doi: 10.1371/journal.pone.0279025 (PMC9833556; doi:10.1371/journal.pone.0279025)

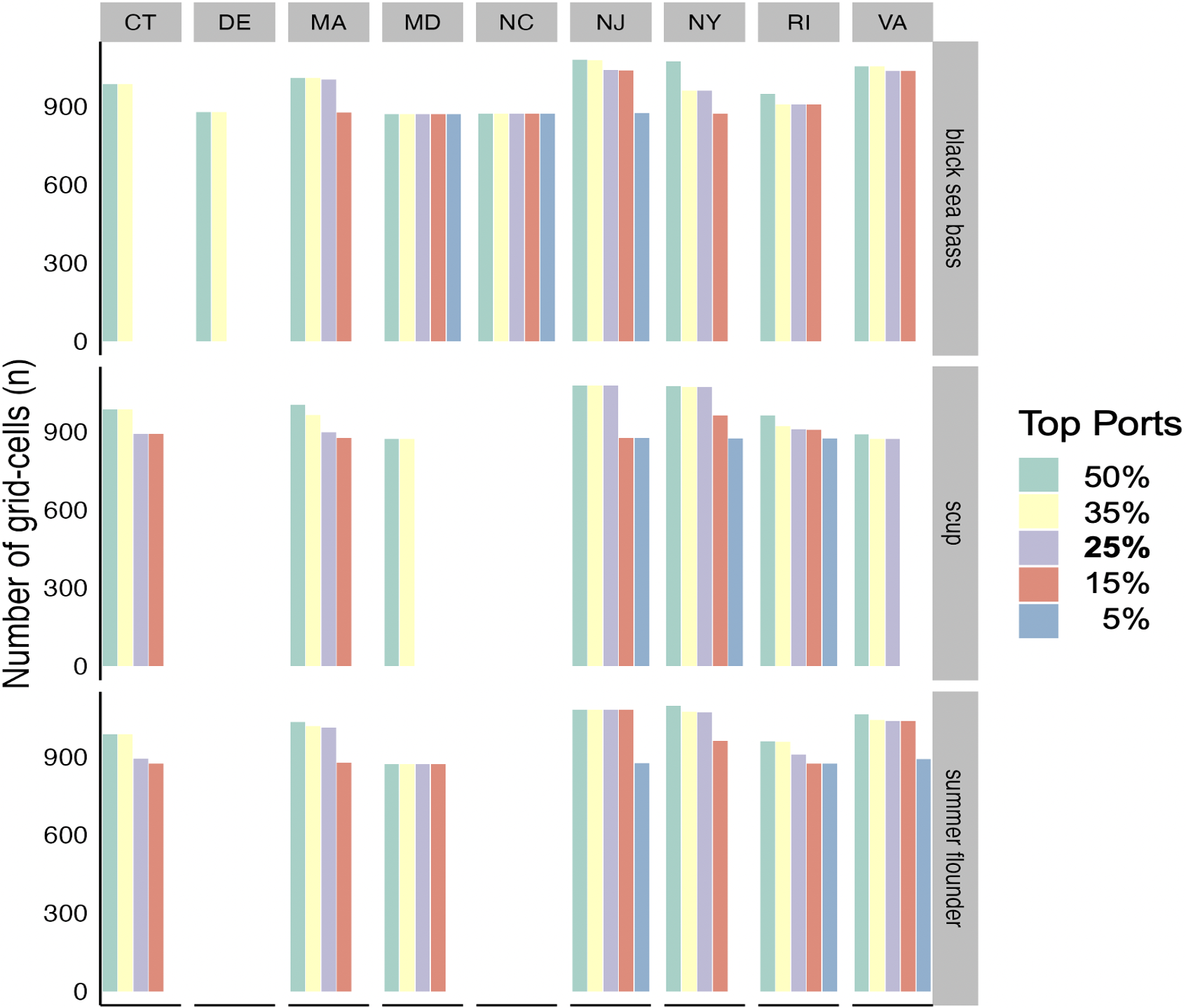

Supplement: S1 Fig — Each color represents the top percentage of ports relative to total landings. This study used the top 25% of ports that landed the greatest number of fish for each stock (In purple and bold in legend). (PNG) [file pone.0279025.s001.png]
