# Supplementary material for: Quantifying fish range shifts across poorly defined management boundaries
Source: PLoS One. 2023 Jan 11;18(1):e0279025. doi: 10.1371/journal.pone.0279025 (PMC9833556; doi:10.1371/journal.pone.0279025)
Supplement: Attachment — Submitted filename: acrossboundaries_rev_two.docx [file pone.0279025.s003.docx]

**Reviewer #2**

**General comments**

This is a really terrific paper. My general suggestions are completely resolvable and are mostly suggestions to improve the paper. Naturally, my minor suggestions are all easily fixed. Except for the one major error I believe I have uncovered, described below, I would recommend this manuscript for publication following only minor revisions.

**Response:** Many thanks for the kind words. We are grateful for the reviewer’s time in revising not only the manuscript but also our code, it certainly makes a big difference, thank you! We believe our mistake was in the description of the methods (and poor code-commenting). What we intended to convey was that we selected the top 25% of ports that landed the most of each stock. This is why the original Fig S1 shows that as you increase that threshold the number of grids decreases (we have reversed this in the new figure). We go over more details below but in short, we have:

- Corrected the text to clearly explain what we intended
- Fixed the legend of supplementary figure S1 to properly represent the percentages
- Corrected the comments in the code to not confuse future potential users

However, unfortunately, there is one major error, I believe, in the analyses that needs to be fixed. This will require redrawing several of the figures and potentially change the results and conclusions, unfortunately. It’s a trivial error, and easy to fix, but sadly it may have big consequences for the paper. Now maybe I’m wrong, I hope I am! But I need to be convinced that I am, because I have thought about this long and hard.

**Response:** We acknowledge that a typo and poor wording of our methods confused the reviewer. What we wanted to convey is that we choose the top *25%* ports that landed each species as a threshold. We have revised this while also including the percentage of total landings that these ports represent. So now we report the number of ports that make the top 25 and the landings that they represent. We have corrected this in the revised manuscript where you will find the following in line 207:

*We selected the main ports landing each stock by sequentially selecting the top 25% ports according to the weight of landings for each stock. Using black sea bass as an example, out of a total of 76 ports with commercial landings, we selected the 19 ports with the largest landings by weight (i.e., (19/76) * 100% = 25% of the ports). This resulted in 19 ports for black sea bass representing 93% of landings, 16 for scup representing 95% of landings, and 23 for flounder representing 97% of landings.*

I believe the fishing port allocation analysis – the analysis to allocate landings to ports up to the 75% threshold of total landings - has been done incorrectly. Thinking about it logically, it doesn’t make sense that the number of total grid cells per state could decrease as the threshold of total landings increases, yet this is what is shown in Supp Fig S1, showing a sensitivity analysis of the 75% threshold versus other thresholds. It shows that as the threshold increases, the number of grid cells per state decreases, in every case. As a thought exercise, let’s say Ports A & B on average land 20% of the total landings, Port C lands 10%, and the rest of the ports land <10% each, adding up to 100% of landings. If you use a ranking system and apply a 50% threshold, then only Ports A-C would get an allocation. But if you apply a 75% threshold, then you start including ports with <10% landings historically, and the total number of ports receiving an allocation goes up. As you increase the threshold, you increase the number of ports contributing to that threshold. However, when you look at Supp FigS1, you can see that the number of grid cells allocated to each state actually *decreases* as the threshold increases. I think that all the analyses based on the fishing port allocation method need to be redone, including most of the figures.

**Response:** The reviewer is correct in their assessment that, as the threshold values increases the number of grid cells decreases. The confusion here is, again, a result of our poor labeling and interpretation, thus, we have revised the figure labeling to say “Top Ports” going from 50 to 5%, and explained in the legend what that meant. Below we provide an explanation of the old figure and a version of the new figure with a new legend.

What the original Figure S1 was showing was a threshold in terms of percentile that limits the catch (not that includes it). Thus, thresholds 50^th^ and 60^th^ represented the top 50% and 40% of ports in terms of catch, 75^th^ represented 25%, 85^th^ represented 15% and 95^th^ represented only the top 5% of ports in terms of higher catches. This is why as the threshold increases, the number of grid cells decreases, as expected by the reviewer. In the figure you see that Connecticut and Delaware have no ports for when you choose the top thresholds (75^th^ – 95^th^) for Black Sea Bass as these represent 25% to 5% of the total landings. We acknowledge this is poorly explained and have made the proper amendments.

The new legend makes the figure more intuitive as the Top 5% of ports represents the smaller number of ports per state while the top 50% includes ports from all states.

**Figure S1. Number of grid cells that each state would have under the ports approach according to the percentage of top fishing ports selected.** Each color represents the top percentage of ports relative to total landings. This study used the top 25% of ports that landed the greatest number of fish for each stock (In purple and bold in legend).

When I go into the GitHub repo and read the R code for the port allocation method, it looks like the threshold ranking has been applied in the inverse. The notation in the code (lines 227-232) says that the top 75% of landings is extracted using “top_frac(0.25…) (line 231). But that code would actually extract the top 25% of landings, not the top 75% of landings, according to the R package documentation: <https://dplyr.tidyverse.org/reference/top_n.html>. This would imply that the results reported in the paper to reflect allocation of 75% of total landings instead reflect allocating only 25%. Likewise, the fractions used in the sensitivity analysis are all in the inverse, as well.

**Response:** Yes, the code is marked at the inverse. A personal note (and admittedly an example of bad code-commenting) of the author that got everyone confused. It is now amended.

Once the analyses are redone, my concern with this threshold is the potential bias against states with lots of smaller ports. The sensitivity analysis is the right way to address this (Fig S1) but it will need to be redone with the right code.

**Response:** Again, we would like to thank the reviewer for taking the time and dedication to revise, nor only the manuscript and supplements, but also our code. We hope that it is now clear that the analysis is correct but the wording of it was misleading.

Also, it doesn’t appear the data are contained in the GitHub repo – all the file pathways point to the lead author's computer filing system.

**Response**: We apologize for this. The data is publicly available from the original sources (e.g., OceanAdapt - <https://oceanadapt.rutgers.edu> and ACCSP - <https://www.accsp.org> . We have uploaded the data to the github repository where the code is found for future reference.

**General suggestions**

**- Introduction**

- I think a more powerful argument in general is that multiple management strategy shifts will be needed to address the challenge of range shifts. The argument in favor of changing methodologies for defining regulatory units and allocations does not depend on other approaches being faulty. “Another tool in the toolbox” is a stronger argument, I think. So I suggest rethinking the section that finds fault with dynamic management rules. The argument there seems to be about difficulty in defining management boundaries, a task also required in the approach suggested here, right?
  - **Response:** Thank you for this comment, we have modified the second paragraph of the introduction to frame the problem to all tools of management, rather than only one. It reads on line 54:

*Ocean management tools at all levels need to incorporate adaptive methods to cope with shifting species distributions [6,7]. For example, marine reserves intended to protect species within their boundaries will need to be composed of networks or be dynamic in nature [8] as part of a strategy to be climate resilient [9]. Moreover, fishing quota allocations based on historical distributions will most likely be outdated as climate changes shift species distributions requiring new allocation methods to follow the fish [10,11]. As such, range shifts in species distribution will pose a challenge for the management of stocks shared across spatial regulatory boundaries [10,12–14]. When a stock falls within two or more spatial regulatory units, managers must often coordinate efforts to maintain sustainability of the (shared) stock [15]. However, there can be a growing disconnect between the region of the ocean where fish are found and the management jurisdictions which hold harvest rights [16]. The requirement for clearly defined resource boundaries is a fundamental principle of managing a common pool natural resource where market failures such as "Tragedy of the Commons"-type negative externalities and "Free Rider"-type positive externalities exist across jurisdictions. Especially when the resource is not managed as a whole but is instead partitioned into multiple jurisdictions. Such market failures generally result in inefficient resource utilization [15].*

- The paragraph starting on line 84 needs a bit of work. I think it’s meant to describe the intersection between spatial regulatory units and allocation in different situations. But the US example isn’t fleshed out – states have the rights to allocate fisheries within their borders, so the regulatory unit is state waters, but you don’t describe the allocation, i.e., to port, or community, as in Latin America. You don’t describe how federal water regulatory units are drawn or right allocated. Transitioning to the seasonal range shifts and migration portion of the paragraph is also a little awkward. Maybe the transition should be, “A challenge for all fixed spatial regulatory units used for allocating fishing rights is that fish distributions may change…” Also, given survey timing is one of the variables of interest in the present analysis, I suggest expanding on line 92-93, the point about seasonality being a key consideration of management, including how survey timing is related (or not) to seasonal distributions.
  - **Response:** Thank you for this observation. We have moved the Latin American examples to the discussion as suggested by the other reviewer. We have expanded in the seasonality part; it now reads in line 91:

*Within the U.S. Atlantic waters, each state allocates fishing rights within its own state waters (i.e., a defined regulatory unit of about 3 nm. from land where states have authority to distribute fisheries rights). However, federal waters (i.e., the 200 nm, EEZ) are not subdivided into sub-regulatory units, except for some specific cases, and in most cases, fishing occurs without state-specific zoning. Nevertheless, a challenge for all fixed spatial regulatory units used for allocating fishing rights in the ocean is that fish distributions may change with seasonality, which is a key consideration in temperate fisheries management [21]. Accounting for seasonality in surveys allows us to understand spatial patterns of reproduction, seasonal phenology and long-term dynamics of a stock [22,23].*

**-Methods**

- I suggest a paragraph at the beginning of the Methods section that describes the current management approach: how quota is set and allocated, using which data (years, seasons) are used, regulatory units, and allocation scheme. Moving the text from lines 170-173, for example, into such a paragraph would be helpful at the beginning of the section.
  - **Response:** Thank you for this comment. The first paragraph of the methods (Fish stock selection) talks about each stock and their management. We have included a sentence specifying the lack of regulatory units in Federal waters, it reads on line 131:

*It is worth mentioning that while the annual coast-wide quota is allocated among states, and each state allocates quota within its own state waters, fish found in the U.S. Federal waters are not allocated to particular states,*

- Fish stock selection, lines 121-122: It is probably worth emphasizing the point that states allocate 100% of the fish stock in their state waters to their state’s vessels, and that it’s the allocation of fish stocks found in federal waters where regulatory units and state allocations really matter, and that is the focus on this paper. That would also eliminate the need to use “in federal waters” throughout. Though, certainly the point should be made that changing fish distributions will certainly also differently affect fisheries in state waters.
  - **Response:** Thank you for this observation**,** we have included this suggestion in the revised manuscript. It now reads on line 131:

*It is worth mentioning that while the annual coast-wide quota is allocated among states, and each state allocates quota within its own state waters, fish found in the U.S. Federal waters are not allocated to particular states. For Federal waters fisheries, an annual coast-wide commercial quota, based on historical landings, was implemented, and since 2003 further allocated to the states of Maine, New Hampshire, Massachusetts, Rhode Island, Connecticut, New York, New Jersey, Delaware, Maryland, Virginia and North Carolina [28] (Table 1). While this work focuses on the consequences of stock shifts to their management within Federal waters, changing fish distributions will also affect fisheries in state waters.*

- Data used: Which years of landings data are used? I suggest stating which years of trawl survey data are used earlier in the paragraph than lines 166-167.
  - **Response:** We have adopted this comment. It now reads on lines 173 and 179:

**Line 173.** *We interpolated the trawl survey data from the observational points to the U.S. EEZ from the southern border of North Carolina to the U.S.-Canadian border for both Fall and Spring seasons covering the time period of 1971 to 2019.*

**Line 179.** *The interpolation was done separately for each season and year.*

- Line 189: How were these years selected as the baseline? Did you do a sensitivity analysis to determine if the selection of those years influences the results?
  - **Response:** We recognize that there are any number of ways to select such years, and that it would probably be a whole management step, itself. Here, we selected these years because *i*) they are the most recent years of data we had available and because they give the longest time period for analysis following the years in which the quota was established. We have included this in the methods, it now reads on line 202:

*We selected these years as they are the most recent years of data available and because they are the furthest away from when the quota was established. However, we recognize that the years of landings data selected might influence the results, and the results should be interpreted with this caveat in mind.*

**- Results**

- Please reconcile what appears to be a contradiction in results about seasonality. The paragraph starting on line 265 reports that seasonality is important in distribution, but the paragraph starting on line 305 reports that the consequences of seasonal differences for allocation are minor.
  - **Response:** Thank you for your observation. Indeed, seasonality plays an important role, but what we wanted to convey on line 305 is that it is less important relative to the method of choosing regulatory areas. We removed the second part of the sentence to avoid confusion. It now reads on line 335:

*Seasonal differences in the proportion of the stock distribution falling within each state's regulatory unit are also evident (Fig. 5).*

**Minor suggestions**

- Line 64: Suggest clarifying the meaning of this sentence by changing to either “have historically resulted” or “historically unsustainable.”
  - **Response:** We have changed the wording to “historically unsustainable”
- Line 78: Regarding reason (i), in the present case, wouldn’t this reason be obsolete?
  - **Response:** No, we mean that regions within the US EEZ are not subdivided, this is the main driver of the article
- Line 80: Change to “EEZs”
  - **Response:** Changed EEZ for EEZs
- Line 80-81: Clarify what is meant by “these boundaries.”
  - **Response:** The sentence now reads “…quantifying fish distributions in relation to spatial regulatory boundaries is difficult…”
- Line 84: “Considering” is vague; can you change to “in association with” or something similar?
  - **Response:** The sentence now reads “Regulatory units can be drawn relative to fishing ports or communities”
- Line 89: Suggest replacing “others” with “other considerations” or “other factors”.
  - **Response:** It now reads “among other considerations”
- Line 108: By “different” do you mean “alternative”? Is one presently implemented and one is not?
  - **Response:** We changed the wording to “alternative”. No approach is currently implemented as far as we know
- Lines 126-129: Make clear that it’s the landings from these historical periods upon which the quotas are based.
  - **Response**: It states on line 134 “An annual coast-wide commercial quota, based on historical landings”
- Table 1: Summer flounder allocations don’t add up to 100%
  - **Response**: This is because we round to only one decimal and Maine, New Hampshire and Delaware have less than 0.1 in terms of quota. We have included the following note in the table head:
    - *Note that summer flounder quota for Maine, New Hampshire and Delaware are 0.05, 0.0005, and 0.02 percent.*
- Lines 144, 148: Change “was” to “were”.
  - **Response:** Changed on revised manuscript
- Lines 217-219: Suggest using “biomass” instead of “trawl survey data”.
  - **Response:** It now reads “biomass”
- Line 226: Suggest replacing “yearly-state estimation” with “year-state estimate”
  - **Response:** It now reads “year-state estimate”
- Lines 227-228: Suggest replacing “then pursue to compare” with “then compared”
  - **Response:** It now reads “then compared”
- Line 226: Why go back as far as 1971 when the earliest data used to set quota for a stock were from 1980?
  - **Response:** Including data from 1971 allows for a better trend observation of historical landings. It does not affect the analysis as we only considered the “last year” and the years where quota was allocated.
- Figure 2: Suggest using common names for the fish to match the text.
  - **Response:** Common names are now presented in all figures
- Figure 3: It would be good to find a way to indicate state names on the figure, given you reference them in the text and there may be international or geographically-challenged American readers. Maybe a key showing just the outline of each state so readers can identify them by shape? Same comment as above about using the common name for the fish.
  - **Response:** We believe our figures are already quite busy and including state names in each one does not help with that. Instead, we have included a new figure to help the reader identify the region where our study focuses. See below

**Figure 1. Location of the states included in this study.** States presented in legend in latitudinal order.
